# The Voluntary Hospitals Conference

**Published:** 1924-07

**Authors:** 


### VOLUNTARY HOSPITALS CONFERENCE.

A Conference between the Voluntary Hospitals Commission and the Local Voluntary Hospital Committees of England and Wales was held on June 18 at the Ministry of Health, Lord Onslow presiding. Mr. Wheatley expressed the Government's appreciation of the excellent work done by the local committees which were set up all over the country a few years ago. A difficult situation arose out of the war, and they felt that no one could have done the work better than the committees with their invaluable local information. Lord Onslow said that the position of the voluntary system was stronger than when Lord Cave's Committee reported. Their present task was to see how far the present accommodation was adequate, and the surer their information the more easy it would be to strengthen and improve the position of the voluntary system.

### The Case of the Tubercular.

A discussion followed on the subject of accommodation in voluntary hospitals and Mr. Hazell, Manchester, referred to cases of surgical tuberculosis requiring prolonged treatment, which occupied beds to the exclusion of other cases, and were, he said, responsible for a large number of persons awaiting admission. He suggested that an alternative method of providing additional beds would be found by adding a surgical wing to every sanatorium. Dr. Menzies said tuberculosis was in the main the responsibility of the Public Health authority, and they should be urged to take that responsibility.

### A Wide View.

Lord Cave thought it would be found that the need for further beds existed more in the case of larger hospitals which served a large surrounding area than in the case of small cottage hospitals. He considered that committees ought to take a wide view and consider not only what a hospital wanted, but what the country wanted in the matter of further accommodation. There had been, until recently, no general system of regarding the hospital question. The time had come when they must apply some system with established principles, and he had a strong feeling that even if they had all the extra beds which individual hospitals were wanting, they might not have done their full duty in connection with the health of the country. There was not enough hospital accommodation in England to-day and there would not be even if we had a few more thousand beds. In the afternoon there was a discussion on the future position of local voluntary hospital committees. Mr. S. R. Lamb spoke of the problem of securing more adequate contributions to hospitals from municipal authorities and insurance organizations, pointing out that business men when approached for definite contributions often raised this question. He had estimated that the hospitals in England and Wales spent £2,334,904 upon treatment for insured persons, and suggested that that sum might very well be regarded as one of the contingencies for which the reserve fund under the National Health Insurance Act, now amounting to £100,000,000, had been set up.

